# Optimization of parameters in coherent spin dynamics of radical pairs in quantum biology

**DOI:** 10.1371/journal.pone.0273404

**Published:** 2023-02-24

**Authors:** Carlos F. Martino, Pablo Jimenez, Max Goldfarb, Ugur G. Abdulla

**Affiliations:** 1 Johns Hopkins University Applied Physics Lab, Laurel, MD, United States of America; 2 Departamento de Física Médica, Instituto Balseiro, San Carlos de Bariloche, Argentina; 3 Department of Mathematical Sciences, Florida Institute of Technology, Melbourne, Fl, United States of America; 4 Analysis & PDE Unit, Okinawa Institute of Science and Technology, Okinawa, Japan; Carl von Ossietzky University of Oldenburg: Carl von Ossietzky Universitat Oldenburg, GERMANY

## Abstract

Identification of the external electromagnetic fields and internal hyperfine parameters which optimize the quantum singlet-triplet yield of simplified radical pairs modeled by Schrödinger system with spin Hamiltonians given by the sum of Zeeman interaction and hyperfine coupling interaction terms are analyzed. A method that combines sensitivity analysis with Tikhonov regularization is implemented. Numerical results demonstrate that the quantum singlet-triplet yield of the radical pair system can be significantly reduced if optimization is pursued simultaneously for both external magnetic fields and internal hyperfine parameters. The results may contribute towards understanding the structure-function relationship of a putative magnetoreceptor to manipulate and enhance quantum coherences at room temperature and leveraging biofidelic function to inspire novel quantum devices.

## Introduction

The idea that quantum effects can be controlled to influence biochemical processes is at the forefront of science [1, 2]. Quantum biology may be thought of as a signature of molecular-level quantum phenomena observed in biological systems at functional, cellular, or organism levels [3–5]. The defining feature of quantum biology is that quantum effects such as coherence and superposition are found at room temperature, in wet environments that typically have lots of motion. The field of quantum biology currently focuses on three main areas of research: photosynthesis, olfaction, [6], and magnetoreception [7–9]. As a point of departure, we concentrate on a less common area, that free-radical production in cellular metabolism may be influenced to some extent by magnetic fields [10]. It is of paramount importance to identify magnetic fields and hyperfine parameters to modulate quantum coherences in a radical pair reaction [11, 12]. In this paper, we identify external electromagnetic field and hyperfine parameters which optimize the quantum singlet-triplet yield of a radical pair system. The rationale is that applications of such integration would enable the control of biological processes that are electromagnetic dependent. We employ the *qlopt* algorithm [13–16] to identify optimal values of 3-dimensional external electromagnetic field vector and 3- or 6-dimensional hyperfine parameter vector which optimize the quantum singlet-triplet yield for the spin dynamics of radical pairs in 8- or 16-dimensional Schrödinger system corresponding to one- and two-proton cases respectively. The method combines ideas of Pontryagin optimization, sensitivity analysis, and Tikhonov regularization. One of the major open problems in optimization of large-scale biological models is the development of the effective global optimization method in a nonlinear and non-convex setting with the least computational cost, which is robust with respect to nonlinearities and scales well with problem size [17]. Currently, such an ideal method does not exist. Deterministic local optimization methods can be used as a global optimization method by embedding a “Multi-start” (MS) strategy into it which facilitates many optimization runs from randomly selected initial parameter guesses [18, 19]. For example, Latin hypercube sampling [20] for partition of the parameter space can be used to guarantee that each parameter estimation iteration starts with an initial guess from a different region in the parameter space. The comparison analysis performed in [17, 19] demonstrates that robust deterministic local optimization methods embedded with MS strategy, and with sharp sensitivity analysis platform are the best candidates for the creation of powerful global optimization methods for large-scale biological and physical models. The comparison analysis of [15, 16] demonstrates the competitiveness and advantage of the *qlopt* algorithm with other most popular local search methods like *lsqnonlin, fmincon, nl2sol*. The main goal of this paper is to develop and adapt *qlopt* method embedded with MS strategy for the quantum optimization in spin dynamics of radical pairs.

The computational analysis demonstrates that the method is very well suited for quantum biology applications. Numerical results demonstrate that the quantum singlet-triplet yield of the radical pair system can be significantly affected if optimal values of external magnetic field and hyperfine parameters are identified.

## Mathematical model of spin dynamics for radical pairs

We consider a spin dynamics of correlated radical pair system, whose law of motion is given by Schrödinger’s equation in the form,

$$\begin{array}{ccc} ı\hbar \frac{d\psi }{dt} & = & H(v)\psi ,\;0\leq t\leq T \end{array}$$ (1)

$$\begin{array}{ccc} \psi (0) & = & \psi_{S}\in C^{n} \end{array}$$ (2)

where

$$\begin{array}{c} \psi \left(t;v\right)=\left(\psi_{1}\left(t;v\right),\ldots ,\psi_{n}\left(t;v\right)\right):\left[0,T\right]\times R^{k}\to C^{n} \end{array}$$

is a state vector, $v\in R^{k}$ is the parameter vector, *T* > 0, ℏ is the Planck constant, *ψ*_*S*_ is a singlet state and

$$\begin{array}{c} H\left(\cdot \right):R^{k}\to C^{n\times n} \end{array}$$

is continuously differentiable matrix-function.

### One-proton model

For a one-proton model (*n* = 8) spin Hamiltonian is given as

$$\begin{array}{c} H=\mu_{B}gS_{1}\cdot u+\mu_{B}gS_{2}\cdot u+\mu_{B}gI_{1}\cdot A_{1}\cdot S_{1}-\frac{ı}{2}(k_{S}P_{S}+k_{T}P_{T}), \end{array}$$ (3)

where **u** = (*u*_*x*_, *u*_*y*_, *u*_*z*_) is the external magnetic field vector, *g* is chosen to be 2 for both radicals, **A**_1_ = (*A*_1*x*_, *A*_1*y*_, *A*_1*z*_) is the anisotropic hyperfine vector for nucleus 1, **I**_1_ = (*I*_1*x*_, *I*_1*y*_, *I*_1*z*_) is the spin operator of nucleus 1, *μ*_*B*_ is the Bohr magneton, **S**_*i*_ = (**S**_*ix*_, **S**_*iy*_, **S**_*iz*_), *i* = 1, 2 are the electron’s spin operators defined as

$$\begin{array}{ccc} S_{1} & = & (\frac{1}{2}\sigma_{x}⊗E_{2}⊗E_{2},\frac{1}{2}\sigma_{y}⊗E_{2}⊗E_{2},\frac{1}{2}\sigma_{z}⊗E_{2}⊗E_{2}), \end{array}$$ (4)

$$\begin{array}{ccc} S_{2} & = & (E_{2}⊗\frac{1}{2}\sigma_{x}⊗E_{2},E_{2}⊗\frac{1}{2}\sigma_{y}⊗E_{2},E_{2}⊗\frac{1}{2}\sigma_{z}⊗E_{2}), \end{array}$$ (5)

where (*σ*_*x*_, *σ*_*y*_, *σ*_*z*_) are the Pauli’s spin matrices acting on the electron’s spins, and **E**_2_ is the 2 x 2 identity matrix. The last term of the spin Hamiltonian is the Haberkorn term “*K*” [21] in an effective non-Hermitian Hamiltonian, where

$$\begin{array}{c} P_{S}=\frac{1}{4}E_{8}-S_{1}\cdot S_{2},\;\;P_{T}=\frac{3}{4}E_{8}+S_{1}\cdot S_{2} \end{array}$$

are projection operators onto the singlet and triplet subspaces respectively. This term reflects the effects of singlet and triplet radical pairs reacting at different rates *k*_*S*_, *k*_*T*_, respectively. The electron-electron exchange, dipolar interaction, and spin relaxation in the radical pair are neglected [22]. Here and the subsequent model without loss of generality, we assume completely static, perfectly aligned ensemble of radical pairs.

The Hamiltonian can be split into **A**-dependent, **u**-dependent and (**u**,**A**)-independent parts, as follows:

$$\begin{array}{c} H=H_{hfi}+H_{z}-ıK, \end{array}$$ (6)

where

$$\begin{array}{c} H_{hfi}=\mu_{B}gA_{1x}I_{1x}S_{1x}+\mu_{B}gA_{1y}I_{1y}S_{1y}+\mu_{B}gA_{1z}I_{1z}S_{1z},\\ H_{z}=\mu_{B}g\left(S_{1x}+S_{2x}\right)u_{x}+\mu_{B}g\left(S_{1y}+S_{2y}\right)u_{y}+\mu_{B}g\left(S_{1z}+S_{2z}\right)u_{z},\\ K=\frac{1}{2}(k_{S}P_{S}+k_{T}P_{T}) \end{array}$$

Spin Hamiltonian **H** is represented by 8 × 8 matrix. We describe explicitly all matrices in S1 Appendix. The Schrödinger system is represented by the system of 8 ordinary differential equations (see S1 Appendix).

### Two-proton model

The following is the spin Hamiltonian in the case of a two-proton model (n = 16):

$$\begin{array}{ccc} H & = & \mu_{B}g\left(S_{1x}+S_{2x}\right)u_{x}+\mu_{B}g\left(S_{1y}+S_{2y}\right)u_{y}+\mu_{B}g\left(S_{1z}+S_{2z}\right)u_{z}\\ & & +\mu_{B}g\left(A_{1x}I_{1x}+A_{2x}I_{2x}\right)S_{1x}+\mu_{B}g\left(A_{1y}I_{1y}+A_{2y}I_{2y}\right)S_{1y}\\ & & +\mu_{B}g\left(A_{1z}I_{1z}+A_{2z}I_{2z}\right)S_{1z}-\frac{ı}{2}(k_{S}P_{S}+k_{T}P_{T}). \end{array}$$ (7)

The model adds the hyperfine interaction of the second proton to the one-proton model (6). Here we assume identical principal axes for the two hyperfine interactions. Spin Hamiltonian **H** is represented by an 16 x 16 matrix.

## Description of the optimization problem and numerical method

Consider the function

$$\begin{array}{c} J\left(v\right)=\frac{k_{S}}{2s}\sum_{l=1}^{s}\int_{0}^{T}\langle \psi^{l}\left(t;v\right)P_{S}\psi^{l}\left(t;v\right)\rangle dt \end{array}$$ (8)

where *ψ*^*l*^(⋅; **v**) is a solution of the Schrödinger system (1), (2) with initial position being at singlet state $\psi_{S}^{l},l=1,\ldots ,s$, $v\in R^{k}$ is a control parameter vector consisting of external magnetic field intensity vector $u\in R^{3}$ and/or internal hyperfine parameter vector $A\in R^{3}$ or $R^{6}$. For the one-proton model (*n* = 8) there are two singlet states (*s* = 2)

$$\begin{array}{c} \psi_{S}^{1}=\frac{e_{3}-e_{5}}{\sqrt{2}},\;\psi_{S}^{2}=\frac{e_{4}-e_{6}}{\sqrt{2}}, \end{array}$$ (9)

and for the two-proton model (*n* = 16) we have four singlet states (*s* = 4)

$$\begin{array}{c} \psi_{S}^{1}=\frac{e_{5}-e_{9}}{\sqrt{2}},\;\psi_{S}^{2}=\frac{e_{6}-e_{10}}{\sqrt{2}},\;\psi_{S}^{3}=\frac{e_{7}-e_{11}}{\sqrt{2}},\;\psi_{S}^{4}=\frac{e_{8}-e_{12}}{\sqrt{2}}, \end{array}$$ (10)

where we adopt the notation {*e*_*j*_} for the standard orthonormal basis in $R^{n}$. The projection operator **P**_*S*_ can be written as

$$\begin{array}{c} P_{S}=\sum_{l=1}^{s}|\psi_{S}^{l}\rangle \langle \psi_{S}^{l}|. \end{array}$$ (11)

Functional J(v) represents quantum singlet yield for the spin dynamics of radical pair system over the time interval [0, *T*]. Our goal is to develop an iterative algorithm for the identification of the optimal value of the control parameter **v** which minimizes the singlet yield over the time interval [0, *T*].

Given an initial guess of the control parameter **v**^0^ we consider iterative algorithm

$$\begin{array}{c} v^{N}=v^{N-1}+\delta v^{N},N=1,2,\ldots \end{array}$$ (12)

To identify an increment *δ* **v**^*N*^ at every iteration first we linearize the state vector

$$\begin{array}{c} \psi^{l}\left(\cdot ;v^{N}\right)=\psi^{l}\left(\cdot ;v^{N-1}\right)+U_{N}^{l}\delta v^{N}+o(|\delta v^{N}\left|\right), \end{array}$$

where *o*(⋅) represents higher than linear order terms, and $U_{N}^{l}$ is the sensitivity matrix

$$\begin{array}{c} U_{N}^{l}\left(t\right)=\frac{\partial \psi^{l}\left(t;v^{N-1}\right)}{\partial v}=\{\frac{\partial \psi_{i}^{l}\left(t;v^{N-1}\right)}{\partial v_{j}},i=1,\ldots ,n,j=1\ldots k\} \end{array}$$ (13)

which satisfies the matrix differential equation

$$\begin{array}{c} ı\hbar \frac{dU_{N}^{l}}{dt}=H\left(v^{N-1}\right)U_{N}^{l}+\Phi ,\;0\leq t\leq T;\;\;U_{N}^{l}\left(0\right)=0 \end{array}$$ (14)

where **Φ** is *n* × *k* matrix with entries

$$\begin{array}{c} \phi_{pq}^{l}=\frac{\partial H^{p}\left(v^{N-1}\right)}{\partial v_{q}}\cdot \psi^{l}\left(t;v^{N-1}\right),\;p=1,\ldots ,n,q=1,\ldots ,k, \end{array}$$

and **H**^*p*^ is a *p*th row vector of **H**.

We identify an increment *δ* **v**^*N*^ as a global minimizer of the function

$$\begin{array}{c} I\left(\delta v\right)=\frac{k_{S}}{2s}\sum_{l=1}^{s}\int_{0}^{T}\langle U_{N}^{l}\delta v+\psi^{l}\left(t;v^{N-1}\right)|P_{S}|U_{N}^{l}\delta v+\psi^{l}\left(t;v^{N-1}\right)\rangle dt, \end{array}$$ (15)

in $R^{k}$. Function (15) represents a quantum singlet yield corresponding to linearized state vector over the time interval [0, *T*]. It is continuously differentiable on $R^{k}$, and

$$\begin{array}{c} DI\left(\delta v\right)=\frac{k_{S}}{s}\sum_{l=1}^{s}\text{Re}\int_{0}^{T}{\left(U_{N}^{l}\right)}^{H}P_{S}|U_{N}^{l}\delta v+\psi^{l}\left(t;v^{N-1}\right)\rangle dt, \end{array}$$ (16)

$$\begin{array}{c} D^{2}I\left(\delta v\right)=\frac{k_{S}}{s}\sum_{l=1}^{s}\int_{0}^{T}{\left(U_{N}^{l}\right)}^{H}P_{S}U_{N}^{l}dt, \end{array}$$ (17)

where ${\left(U_{N}^{l}\right)}^{H}$ is a conjugate transpose of the sensitivity matrix $U_{N}^{l}$. The matrix ${\left(U_{N}^{l}\right)}^{H}P_{S}U_{N}^{l}$ is Hermitian. Indeed, if $u_{ij}^{l}$ is its entry on *i*^*th*^ row and *j*^*th*^ column, and *e*_*i*_ is a unit *x*_*i*_-vector in $R^{k}$, then we have

$$\begin{array}{c} u_{ij}^{l}=\int_{0}^{T}\langle e_{i}|{\left(U_{N}^{l}\right)}^{H}P_{S}U_{N}^{l}|e_{j}\rangle \;dt=\int_{0}^{T}\langle U_{N}^{l}e_{i}|P_{S}|U_{N}^{l}e_{j}\rangle \;dt\\ =\int_{0}^{T}{\langle (U_{N}^{l})}^{i}|P_{S}|{\left(U_{N}^{l}\right)}^{j}\rangle \;dt=\int_{0}^{T}\bar{{\langle (U_{N}^{l})}^{j}|P_{S}|{\left(U_{N}^{l}\right)}^{i}}\rangle \;dt=\bar{u_{ji}^{l}}. \end{array}$$ (18)

Since

$$\begin{array}{c} \int_{0}^{T}\langle \psi |{\left(U_{N}^{l}\right)}^{H}P_{S}U_{N}^{l}|\psi \rangle \;dt=\sum_{m=1}^{s}\int_{0}^{T}{\left|\langle \psi_{S}^{m}|U_{N}^{l}\psi \rangle \right|}^{2}\;dt\geq 0, \end{array}$$

it follows that the Hermitian matrix $\int_{0}^{T}{\left(U_{N}^{l}\right)}^{H}P_{S}U_{N}^{l}\;dt$ is positive semi-definite. Thererfore, the minimum point *δ* **v**^*N*^ satisfies the following system of *k* linear algebraic equations

Aδv=P, (19)

where

$$\begin{array}{c} A=\sum_{l=1}^{s}\text{Re}\int_{0}^{T}{\left(U_{N}^{l}\right)}^{H}P_{S}U_{N}^{l}\;dt, \end{array}$$ (20)

and

$$\begin{array}{c} P=-\sum_{l=1}^{s}\text{Re}\int_{0}^{T}{\left(U_{N}^{l}\right)}^{H}P_{S}|\psi^{l}\left(t,v^{N-1}\right)\rangle \;dt \end{array}$$ (21)

### Optimality condition via Gramian matrix of $\frac{\partial }{\partial v_{i}}\left(\begin{array}{c} \psi_{3}-\psi_{5}\\ \psi_{4}-\psi_{6} \end{array}\right)$ for the one-proton model

Let $L_{2}^{2}\left(0,T;C^{2}\right)$ be an Hilbert space of Lebesgue measurable vector functions $\Psi :\left(0,T\right)\to C^{2}$ with inner product

$$\begin{array}{c} \langle \Psi_{1}|{\Psi_{2}\rangle }_{L_{2}^{2}\left(0,T;C^{2}\right)}=\int_{0}^{T}\Psi_{1}^{H}\Psi_{2}\;dt \end{array}$$

Let $\Psi_{N}^{l},{\left(V_{N}^{l}\right)}^{i}\in L_{2}^{2}\left(0,T;C^{2}\right)$ are defined as

$$\begin{array}{c} \Psi_{N}^{l}:=(\begin{array}{c} \psi_{3}^{l}\left(\cdot ;v_{N-1}\right)-\psi_{5}^{l}\left(\cdot ;v_{N-1}\right)\\ \psi_{4}^{l}\left(\cdot ;v_{N-1}\right)-\psi_{6}^{l}\left(\cdot ;v_{N-1}\right) \end{array}),\;{\left(V_{N}^{l}\right)}^{i}:=\frac{\partial \Psi_{N}^{l}}{\partial v_{i}},l=1,\ldots ,s;\;i=1,\ldots ,k, \end{array}$$

and

$$\begin{array}{c} V_{N}^{l}:=\frac{\partial \Psi_{N}^{l}}{\partial v} \end{array}$$

is a corresponding 2×*k* sensitivity matrix with columns ${\left(V_{N}^{l}\right)}^{i}$. With this notations, we can easily deduce that

$$\begin{array}{c} \int_{0}^{T}{\left(U_{N}^{l}\right)}^{H}P_{S}U_{N}^{l}\;dt=\frac{1}{2}\int_{0}^{T}{\left(V_{N}^{l}\right)}^{H}V_{N}^{l}\;dt \end{array}$$ (22)

$$\begin{array}{c} -\int_{0}^{T}{\left(U_{N}^{l}\right)}^{H}P_{S}|\psi^{l}\left(t,v^{N-1}\right)\rangle \;dt=-\frac{1}{2}\int_{0}^{T}{\left(V_{N}^{l}\right)}^{H}|\Psi_{N}^{l}\rangle \;dt, \end{array}$$ (23)

where *k* × *k* matrix $\int_{0}^{T}{\left(V_{N}^{l}\right)}^{H}V_{N}^{l}\;dt$ is the Gram matrix of vectors ${\left(V_{N}^{l}\right)}^{i}$, *i* = 1,…, *k* in $L_{2}^{2}\left(0,T;C^{2}\right)$, i.e.

$$\begin{array}{c} \int_{0}^{T}{\left(V_{N}^{l}\right)}^{H}V_{N}^{l}\;dt={\left(a_{ij}^{l}\right)}_{i,j=1}^{k},\;a_{ij}^{l}={\langle (V_{N}^{l})}^{i}|{{\left(V_{N}^{l}\right)}^{j}\rangle }_{L_{2}^{2}\left(0,T;C^{2}\right)}, \end{array}$$ (24)

$$\begin{array}{c} \int_{0}^{T}{\left(V_{N}^{l}\right)}^{H}|\Psi_{N}^{l}\rangle \;dt={\left(p_{i}^{l}\right)}_{i=1}^{k},\;p_{i}^{l}=\langle {\left(V_{N}^{l}\right)}^{i}|{\Psi_{N}^{l}\rangle }_{L_{2}^{2}\left(0,T;C^{2}\right)}. \end{array}$$ (25)

Hence, our optimality system is (19), with

$$\begin{array}{c} A=\sum_{l=1}^{s}\frac{1}{2}\text{Re}\int_{0}^{T}{\left(V_{N}^{l}\right)}^{H}V_{N}^{l}\;dt,\;\;P=-\sum_{l=1}^{s}\frac{1}{2}\text{Re}\int_{0}^{T}{\left(V_{N}^{l}\right)}^{H}|\Psi_{N}^{l}\rangle \;dt \end{array}$$ (26)

To transform it further, introduce an Hilbert space $L_{2}^{4}\left(0,T;R^{4}\right)$ of Lebesgue measurable vector functions $\Phi :\left(0,T\right)\to R^{4}$ with inner product

$$\begin{array}{c} \langle \Phi_{1}|{\Phi_{2}\rangle }_{L_{2}^{4}\left(0,T;R^{4}\right)}=\int_{0}^{T}\Phi_{1}^{T}\Phi_{2}\;dt \end{array}$$

Let $\Phi_{N}^{l},{\left(W_{N}^{l}\right)}^{i}\in L_{2}^{4}\left(0,T;R^{4}\right)$ are defined as

$$\begin{array}{c} \Phi_{N}^{l}:=(\begin{array}{c} \text{Re}(\psi_{3}^{l}\left(\cdot ;v_{N-1}\right)-\psi_{5}^{l}\left(\cdot ;v_{N-1}\right))\\ \text{Im}(\psi_{3}^{l}\left(\cdot ;v_{N-1}\right)-\psi_{5}^{l}\left(\cdot ;v_{N-1}\right))\\ \text{Re}(\psi_{4}^{l}\left(\cdot ;v_{N-1}\right)-\psi_{6}^{l}\left(\cdot ;v_{N-1}\right))\\ \text{Im}(\psi_{4}^{l}\left(\cdot ;v_{N-1}\right)-\psi_{6}^{l}\left(\cdot ;v_{N-1}\right)) \end{array}),{\left(W_{N}^{l}\right)}^{i}:=\frac{\partial \Phi_{N}^{l}}{\partial v_{i}},i=1,\ldots ,k \end{array}$$

and

$$\begin{array}{c} W_{N}^{l}:=\frac{\partial \Phi_{N}^{l}}{\partial v} \end{array}$$

is a corresponding 4 × *k* sensitivity matrix with columns ${\left(W_{N}^{l}\right)}^{i}$. We now deduce that

$$\begin{array}{c} \text{Re}\int_{0}^{T}{\left(U_{N}^{l}\right)}^{H}P_{S}U_{N}^{l}\;dt=\frac{1}{2}\text{Re}\int_{0}^{T}{\left(V_{N}^{l}\right)}^{H}V_{N}^{l}\;dt=\frac{1}{2}\int_{0}^{T}{\left(W_{N}^{l}\right)}^{T}W_{N}^{l}\;dt\\ -\text{Re}\int_{0}^{T}{\left(U_{N}^{l}\right)}^{H}P_{S}|\psi^{l}\left(t,v^{N-1}\right)\;dt=-\text{Re}\frac{1}{2}\int_{0}^{T}{\left(V_{N}^{l}\right)}^{H}|\Psi_{N}^{l}\rangle \;dt\\ =-\frac{1}{2}\int_{0}^{T}{\left(W_{N}^{l}\right)}^{T}|\Phi_{N}^{l}\rangle \;dt, \end{array}$$ (27)

where *k* × *k* matrix $\int_{0}^{T}{\left(W_{N}^{l}\right)}^{T}W_{N}^{l}\;dt$ is the Gram matrix of vectors ${\left(W_{N}^{l}\right)}^{i},i=1,\ldots ,k$ in $L_{2}^{4}\left(0,T;R^{4}\right)$, i.e.

$$\begin{array}{c} \int_{0}^{T}{\left(W_{N}^{l}\right)}^{T}W_{N}^{l}\;dt={\left(w_{ij}^{l}\right)}_{i,j=1}^{k},\;w_{ij}^{l}=\langle {\left(W_{N}^{l}\right)}^{i}|{{\left(W_{N}^{l}\right)}^{j}\rangle }_{L_{2}^{4}\left(0,T;R^{4}\right)}, \end{array}$$ (28)

$$\begin{array}{c} P_{l}:=\int_{0}^{T}{\left(W_{N}^{l}\right)}^{T}|\Phi_{N}^{l}\rangle \;dt={\left(f_{i}^{l}\right)}_{i=1}^{k},\;f_{i}^{l}=\langle {\left(W_{N}^{l}\right)}^{i}|{\Phi_{N}^{l}\rangle }_{L_{2}^{4}\left(0,T;R^{4}\right)}. \end{array}$$ (29)

Hence, our optimality system can be written as (19), with

$$\begin{array}{c} A=\sum_{l=1}^{s}A_{l},\;P=\sum_{l=1}^{s}P_{l}, \end{array}$$ (30)

where

$$\begin{array}{c} A_{l}=\int_{0}^{T}{\left(W_{N}^{l}\right)}^{T}W_{N}^{l}\;dt,\;\;P=-\int_{0}^{T}{\left(W_{N}^{l}\right)}^{T}|\Phi_{N}^{l}\rangle \;dt \end{array}$$

We have

$$\begin{array}{c} detA_{l}=\Gamma \left({\left(W_{N}^{l}\right)}^{1},\ldots ,{\left(W_{N}^{l}\right)}^{k}\right):=det\int_{0}^{T}{\left(W_{N}^{l}\right)}^{T}W_{N}^{l}\;dt, \end{array}$$ (31)

where $\Gamma ({\left(W_{N}^{l}\right)}^{1},\ldots ,{\left(W_{N}^{l}\right)}^{k})$ is a Gram determinant of vectors $\left\{{\left(W_{N}^{l}\right)}^{i},i=1,\ldots ,k\right\}\subset L_{2}^{4}\left(0,T;R^{4}\right)$. It is well known [23] that

$$\begin{array}{c} detA_{l}=\Gamma \left({\left(W_{N}^{l}\right)}^{1},\ldots ,{\left(W_{N}^{l}\right)}^{k}\right)\geq 0, \end{array}$$ (32)

and it is positive, that is to say, **A**_*l*_ is a non-singular matrix, if and only if the vectors ${\left(W_{N}^{l}\right)}^{i},i=1,\ldots ,k$ are linearly independent in $L_{2}^{4}\left(0,T;R^{4}\right)$. Applying Minkowski’s Determinant Theorem [24], from (30) we deduce the low bound for *det* **A**:

$$\begin{array}{c} detA\geq {\left[\sum_{l=1}^{s}{\left(detA_{l}\right)}^{\frac{1}{k}}\right]}^{k} \end{array}$$ (33)

The estimation (33) implies that the matrix **A** is non-singular, if *det* **A**_*l*_ > 0 for some *l* ∈ {1,…, *s*}. Hence, we established the following

**Theorem 1** *Optimality system* (19) *has a unique solution, which is a unique global minimizer of the function* (15) *in* $R^{k}$ *if the functions* ${\left(W_{N}^{l}\right)}^{i},i=1,\ldots ,k$ *are linearly independent in* $L_{2}^{4}\left(0,T;R^{4}\right)$ *for some singlet state* $\psi_{S}^{l},\;l\in \left\{1,\ldots ,s\right\}$.

### Optimality condition via Gramian matrix of $\frac{\partial }{\partial v_{i}}\left(\begin{array}{c} \psi_{5}-\psi_{9}\\ \psi_{6}-\psi_{10}\\ \psi_{7}-\psi_{11}\\ \psi_{8}-\psi_{12} \end{array}\right)$ for the two-proton model

Let $\Psi_{N}^{l},{\left(V_{N}^{l}\right)}^{i}\in L_{2}^{4}\left(0,T;C^{4}\right)$ are defined as

$$\Psi_{N}^{l}:=(\begin{array}{c} \psi_{5}^{l}\left(\cdot ;v_{N-1}\right)-\psi_{9}^{l}\left(\cdot ;v_{N-1}\right)\\ \psi_{6}^{l}\left(\cdot ;v_{N-1}\right)-\psi_{10}^{l}\left(\cdot ;v_{N-1}\right)\\ \psi_{7}^{l}\left(\cdot ;v_{N-1}\right)-\psi_{11}^{l}\left(\cdot ;v_{N-1}\right)\\ \psi_{8}^{l}\left(\cdot ;v_{N-1}\right)-\psi_{12}^{l}\left(\cdot ;v_{N-1}\right) \end{array}),\;{\left(V_{N}^{l}\right)}^{i}:=\frac{\partial \Psi_{N}^{l}}{\partial v_{i}},i=1,\ldots ,k$$

and

$$\begin{array}{c} V_{N}^{l}≔\frac{\partial \Psi_{N}^{l}}{\partial v} \end{array}$$

is a corresponding 4 × *k* sensitivity matrix with columns ${\left(V_{N}^{l}\right)}^{i}$. With this notations, we can easily deduce (22)-(26), where the corresponding Hilbert space is $L_{2}^{4}\left(0,T;C^{4}\right)$.

To transform it further, let $\Phi_{N}^{l},{\left(W_{N}^{l}\right)}^{i}\in L_{2}^{8}\left(0,T;R^{8}\right)$ are defined as

$$\Phi_{N}^{l}≔(\begin{array}{c} \text{Re}(\psi_{5}^{l}\left(\cdot ;v_{N-1}\right)-\psi_{9}^{l}\left(\cdot ;v_{N-1}\right))\\ \text{Im}(\psi_{5}^{l}\left(\cdot ;v_{N-1}\right)-\psi_{9}^{l}\left(\cdot ;v_{N-1}\right))\\ \text{Re}(\psi_{6}^{l}\left(\cdot ;v_{N-1}\right)-\psi_{10}^{l}\left(\cdot ;v_{N-1}\right))\\ \text{Im}(\psi_{6}^{l}\left(\cdot ;v_{N-1}\right)-\psi_{10}^{l}\left(\cdot ;v_{N-1}\right))\\ \text{Re}(\psi_{7}^{l}\left(\cdot ;v_{N-1}\right)-\psi_{11}^{l}\left(\cdot ;v_{N-1}\right))\\ \text{Im}(\psi_{7}^{l}\left(\cdot ;v_{N-1}\right)-\psi_{11}^{l}\left(\cdot ;v_{N-1}\right))\\ \text{Re}(\psi_{8}^{l}\left(\cdot ;v_{N-1}\right)-\psi_{12}^{l}\left(\cdot ;v_{N-1}\right))\\ \text{Im}(\psi_{8}^{l}\left(\cdot ;v_{N-1}\right)-\psi_{12}^{l}\left(\cdot ;v_{N-1}\right)) \end{array}),\;{\left(W_{N}^{l}\right)}^{i}≔\frac{\partial \Phi_{N}^{l}}{\partial v_{i}},i=1,\ldots ,k$$

and

$$\begin{array}{c} W_{N}^{l}≔\frac{\partial \Phi_{N}^{l}}{\partial v} \end{array}$$

is a corresponding 8 × *k* sensitivity matrix with columns ${\left(W_{N}^{l}\right)}^{i}$. With this notation we deduce (27)-(33) with corresponding Hilbert space $L_{2}^{8}\left(0,T;R^{8}\right)$.

Hence, Theorem 1 applies to two-proton model with corresponding Hilbert space replaced with $L_{2}^{8}\left(0,T;R^{8}\right)$.

### Algorithm

The following iterative method is suggested for the identification of the optimal value of the parameter **v**:

1. Initialize **v**^0^ and set *N* = 1.
2. Find *ψ*^*l*^(*t*;**v**^*N*−1^) and the sensitivity matrices $U_{N}^{l}$ for *l* = 1,…, *s* by solving systems of differential Eqs (1) and (2) with $\psi_{S}=\psi_{S}^{l}$, and (14) respectively.
3. Find state vector $\Phi_{N}^{l}$, sensitivity vectors ${\left(W_{N}^{l}\right)}^{i},i=1,\ldots ,k$, and identify the matrix **A**, and the vector **P** from (30).
4. Find *δ* **v**^*N*^ by solving the linear algebraic equations system (19) and update the new value **v**^*N*^ by (12).
5. If necessary accuracy is achieved, then terminate the process, otherwise replace *N* with *N*+ 1 and go to Step 2. As termination criteria, the smallest of either of the expressions
  $$\left|v^{N-1}-v^{N}|,\;I(\delta v\right)$$ (34)
  can be used.

### Regularization

In general, the matrix **A** may be ill-conditioned, and to solve the ill-conditioned problem (19) we implement Tikhonov regularization. To derive the gradient update needed to apply Tikhonov regularization, we consider the cost functional

$$\begin{array}{c} I\left(\delta v\right)=\frac{k_{S}}{2s}\sum_{l=1}^{s}\int_{0}^{T}\langle U_{N}^{l}\delta v+\psi^{l}\left(t;v^{N-1}\right)|P_{S}|U_{N}^{l}\delta v+\psi^{l}\left(t;v^{N-1}\right)\rangle dt+\frac{\lambda T}{2}{\left|\delta v\right|}^{2}, \end{array}$$

where λ > 0 is a regularization parameter scaled according to the final moment *T*. This yields the following linear system instead of (19)

$$\begin{array}{c} (A+\lambda TE_{k})\delta v=P, \end{array}$$ (35)

where **A** and **P** are defined as in (30), and **E**_*k*_ is the *k* × *k* identity matrix.

## Results

### One-proton model

#### Validation of radical pair model

We validate the model with the case of a radical pair with a single spin-1/2 nucleus and a static magnetic field in the direction $\hat{u}=\frac{u}{∥u∥}$, where **u** = (−0.2, −0.97, 0.11)*μT* and *T* = 14*μs*. In other words, the norm of the field changed while the direction is kept constant. The final moment *T* is chosen such that the product yield J corresponding to Hamiltonian for the classical resonance at 1.4 MHz, and rate constants *k* = 0.5 will be in a 10^−4^-neighborhood of its asymptotic limit as *T* → ∞. Hyperfine constants are chosen as *A*_1*x*_ = −0.234, *A*_1*y*_ = −0.234, *A*_1*z*_ = 0.117 all in mT; the constants were chosen in the spirit of [25]. For this and the subsequent model, reaction rates *k*_*S*_, *k*_*T*_ are set equal with value of $k_{S}^{-1}=2\mu s$.

$$\begin{array}{ccc} H & = & \mu_{B}g\left(S_{1z}+S_{2z}\right)u_{z}+\mu_{B}gI_{1x}A_{1x}S_{1x}+\mu_{B}gI_{1y}A_{1y}S_{1y}+\mu_{B}gI_{1z}A_{1z}S_{1z}\\ & & -\frac{ı}{2}(k_{S}P_{S}+k_{T}P_{T}), \end{array}$$ (36)

where **I**_1_, **S**_1_ and **S**_2_ are the spin angular momentum operators of the nucleus and two electrons.

**Fig 1:**
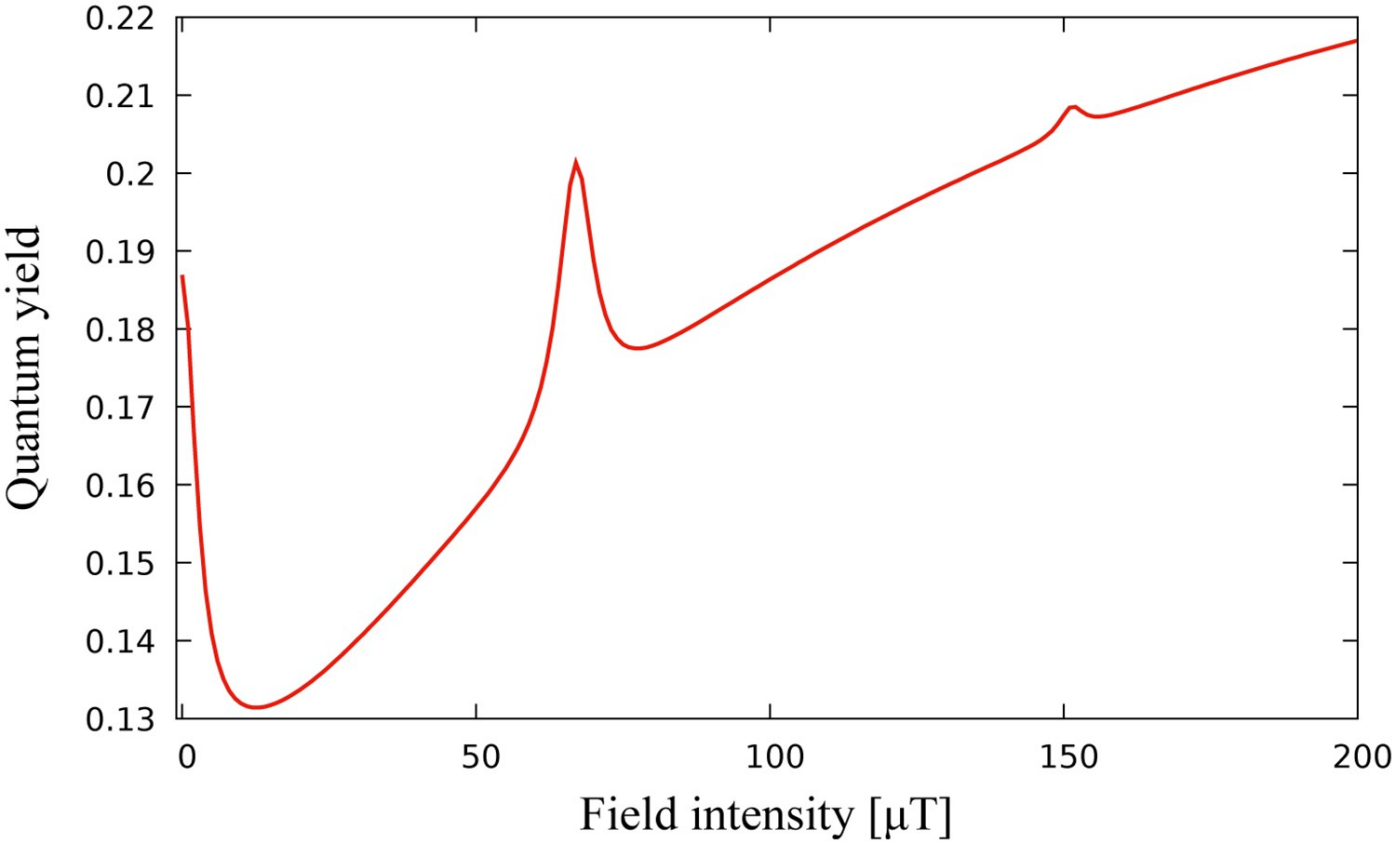
Dependence of the singlet yield on the strength of the applied magnetic field. Singlet yield is minimized for low level magnetic fields. Hyperfine constants are chosen as *A*_1*x*_ = −0.234, *A*_1*y*_ = −0.234, *A*_1*z*_ = 0.117 all in mT. These values are chosen in the spirit of [25].

Fig 1 shows the static magnetic field effect on the singlet quantum yield of a one-proton radical pair, calculated using (8). A striking feature of Fig 1 is the drop of the quantum yield J produced at low level fields.

#### Identification of magnetic parameters to minimize quantum yield

**Fig 2:**
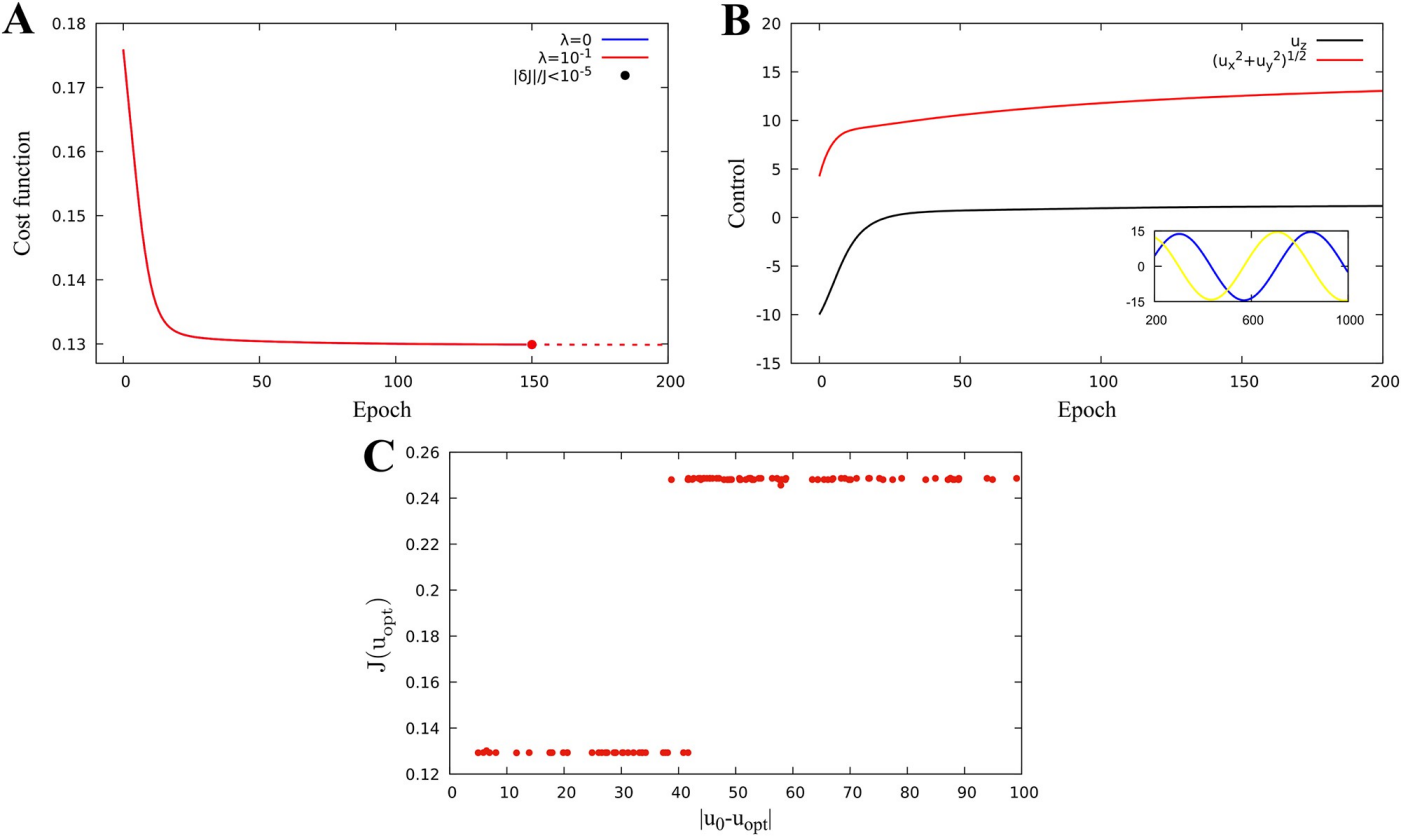
One proton model field optimization. Identification of magnetic parameters for a one-proton model with hyperfine constants *A*_1*x*_ = −0.234, *A*_1*y*_ = −0.234, and *A*_1*z*_ = 0.117. Epoch stands for iteration parameter n. **A:** Minimization of the cost function. **B:** Magnetic parameter evolution. **C:** Final value of the cost function versus distance of initial iteration from the optimal parameter.

We consider optimization problem when *k* = 3, $v=u=\left(u_{x},u_{y},u_{z}\right)\in R^{3}$, i.e. we search for the external magnetic field vector which minimizes the singlet quantum yield. Fig 2 demonstrate the results of application of the algorithm. The iterative sequence converges with respect to cost function (Fig 2A), as well as with respect to control parameter (Fig 2B). Fig 2B demonstrates that the minimum of the quantum singlet yield has a radial symmetry with respect to (*u*_*x*_, *u*_*y*_) component of the external magnetic field vector. Therefore, once iterative process achieves the minimum values of *u*_*z*_ and ${\left(u_{x}^{2}+u_{y}^{2}\right)}^{\frac{1}{2}}$, the component (*u*_*x*_, *u*_*y*_) stays on the circle of minimum radius by producing periodic iteration of *u*_*x*_ and *u*_*y*_ shown in the inset of Fig 2B. Fig 2C demonstrates the convergence range of the optimal solution *u*_*opt*_ from Fig 2B. Convergence range is defined as a neighbourhood of optimal solution *u*_*opt*_ in $R^{3}$ such that for any *u*_0_ chosen from it, the sequence *u*_*N*_ constructed according to algorithm converges to *u*_*opt*_. The low branch of the graph in Fig 2C corresponds to the initial guess of magnetic field vector satisfying |*u*_0_ − *u*_*opt*_| < 42, whereas the upper branch corresponds to the case |*u*_0_ − *u*_*opt*_| ≥ 42. Hence, the quantum yield converges to the minimum value J=0.13 represented by the dose-response model in Fig 1, if the initial value of the magnetic field vector satisfies the inequality |*u*_0_ − *u*_*opt*_| < 42. If |*u*_0_−*u*_*opt*_| ≥ 42 it still converges, but to a higher local minimum value of 0.25.

#### Identification of hyperfine parameters to minimize quantum yield

**Fig 3:**
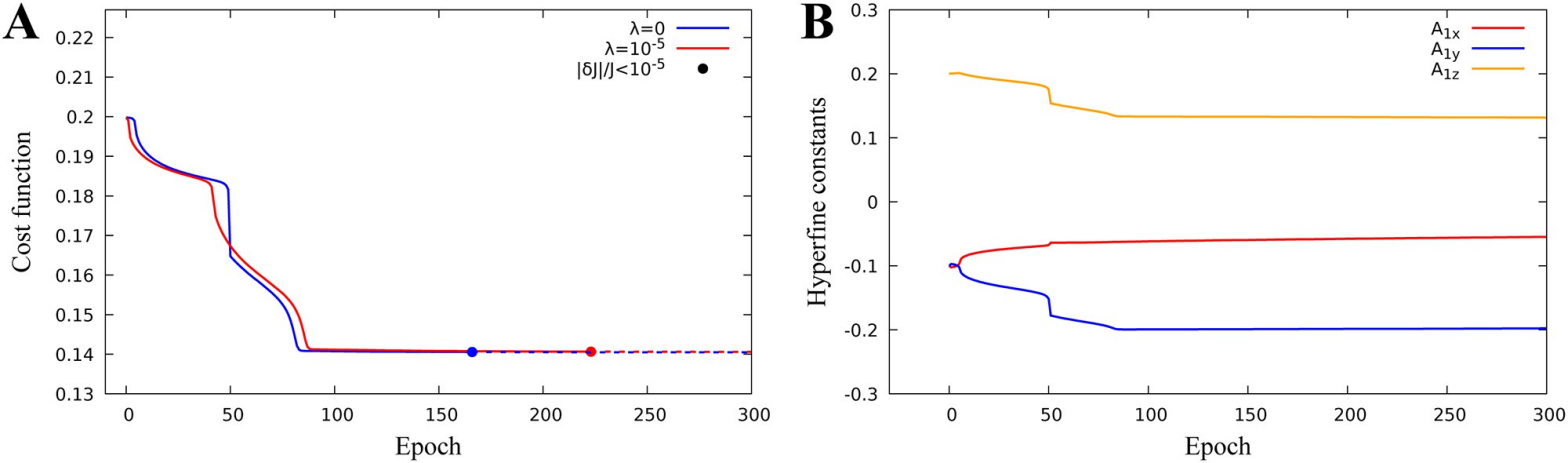
One proton model hyperfine optimization. Identification of hyperfine paramters for a one-proton model. The cost function minimum J=0.14 is reached for diagonal hyperfine constants *A*_1*x*_ = −0.054, *A*_1*y*_ = −0.198 and *A*_1*z*_ = 0.131 and using a regularization parameter λ = 10^−5^. **A:** Cost function minimization with or without regularization. **B:** Hyperfine parameter evolution.

We consider optimization problem when *k* = 3, $v=A_{1}=\left(A_{1x},A_{1y},A_{1z}\right)\in R^{3}$, i.e. we search for the hyperfine parameters which minimize the singlet quantum yield. We fix the magnetic field parameter at **u** = (30, 10, 40)*μT* corresponding to the Earth’s magnetic field. Minimization of cost functional and identified hyperfine parameters are shown in (Fig 3).

#### Identification of combined parameters to minimize quantum yield

**Fig 4:**
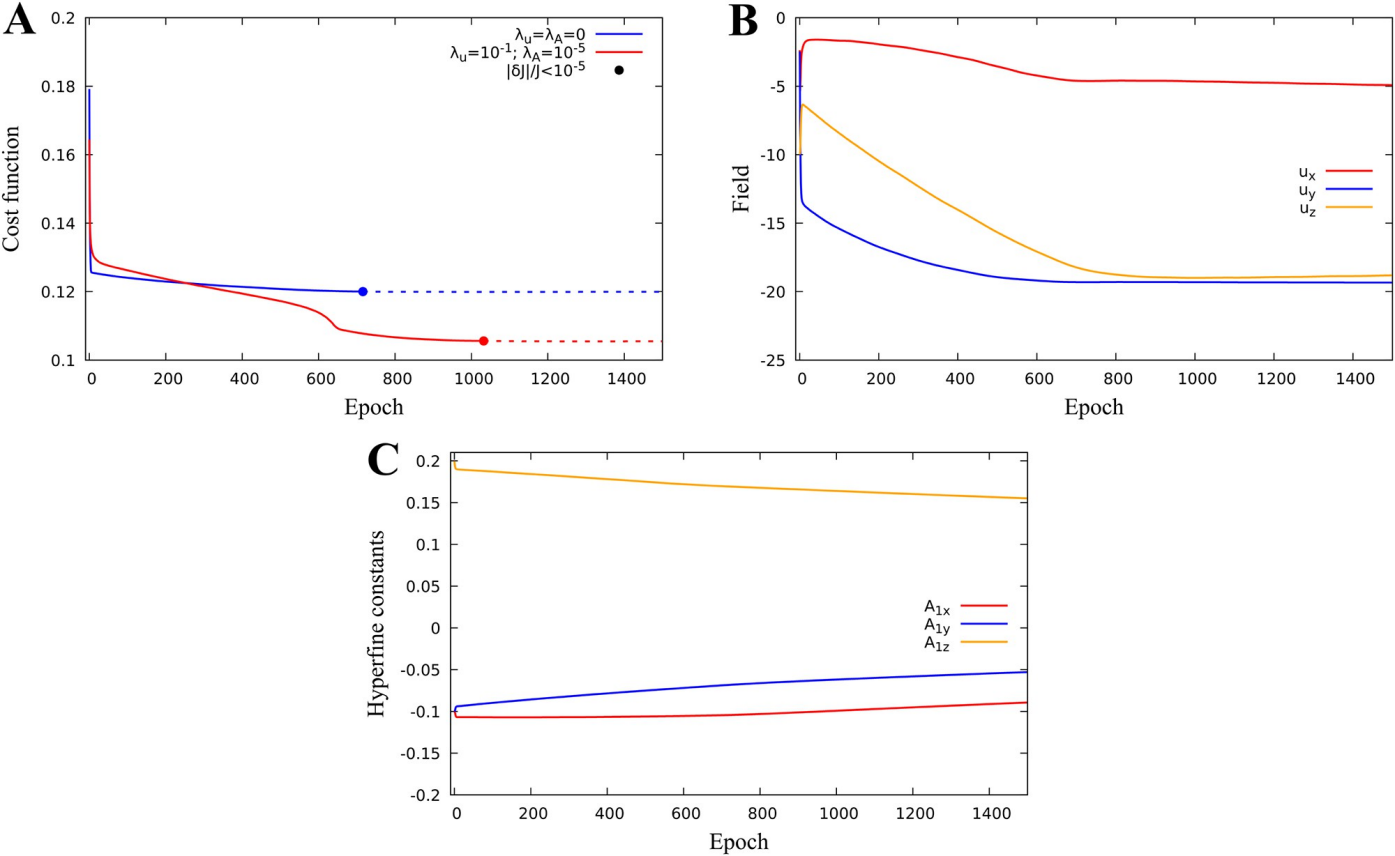
Identification of the magnetic field and hyperfine parameters for a one-proton model. Identification of the magnetic field and hyperfine parameters for a one-proton model. The cost function minimum J=0.106 is reached for hyperfine constants *A*_1*x*_ = −0.089, *A*_1*y*_ = −0.053, *A*_1*z*_ = 0.155 for a magnetic field (*u*_*x*_, *u*_*y*_, *u*_*z*_) = (-4.92, -19.33, -18.81) *μT*. **A:** Cost function minimization with or without regularization. **B:** Magnetic field parameter evolution. **C:** Hyperfine parameter evolution.

We now consider optimization when *k* = 6, $v=\left(u,A_{1}\right)\in R^{6}$, i.e. we search for both external magnetic field and hyperfine parameters which minimize the singlet quantum yield. Remarkably, joint optimization reduces the minimum of the quantum yield to its minimum value 0.12 without regularization (blue graph in Fig 4A), and to 0.106 with regularization (red graph in Fig 4A) which is significantly smaller than its minimum value in the dose-response model.

### Two-proton model

#### Validation

**Fig 5:**
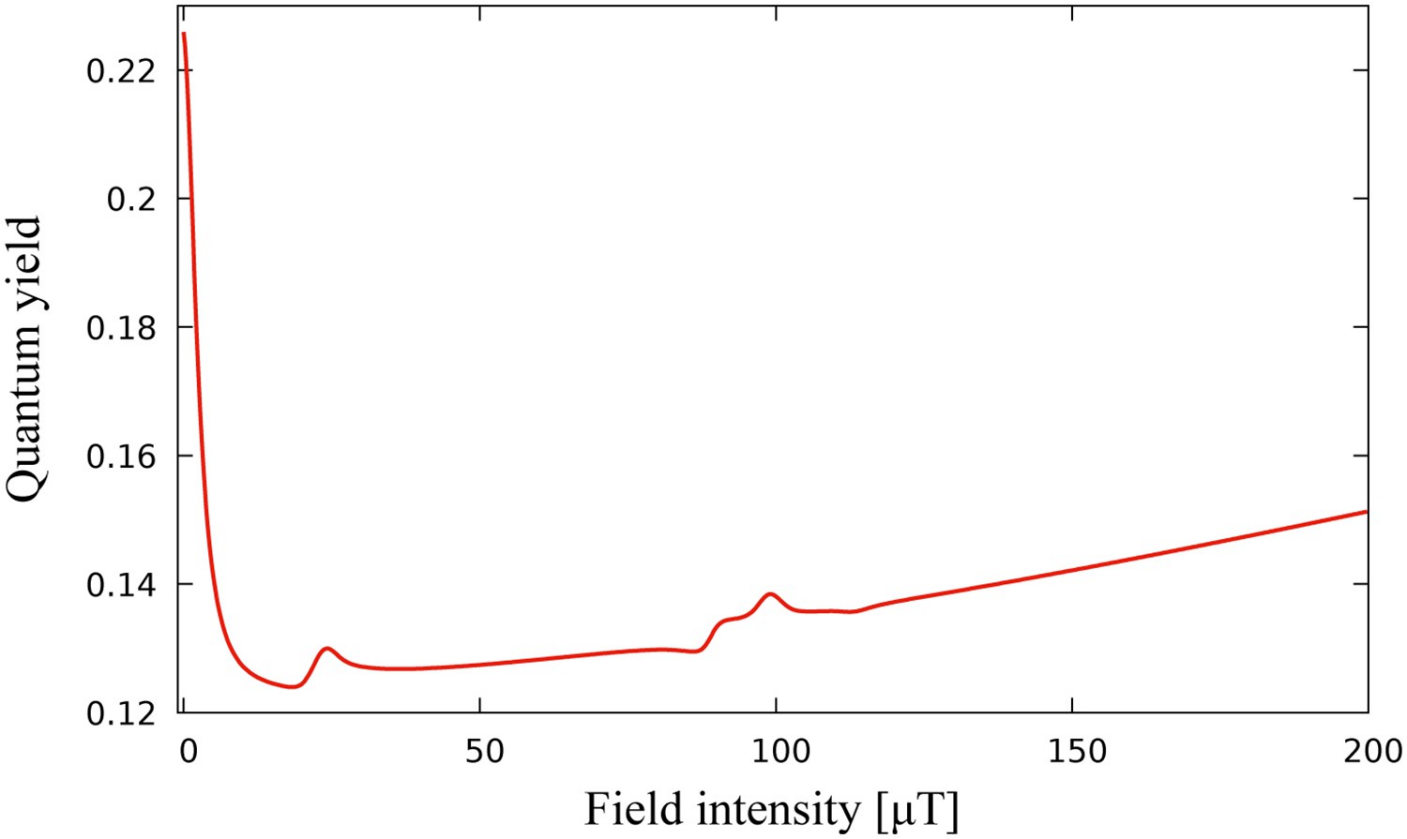
Quantum yield as a function of magnetic field for two-proton model. Hyperfine constants are chosen as *A*_1*x*_ = 0.03, *A*_1*y*_ = −0.64, *A*_1*z*_ = 0.17, *A*_2*x*_ = −0.10, *A*_2*y*_ = 0.0, *A*_2*z*_ = 0.05 all in mT.

As before, we validate the model with the case of a radical pair with a two spin-1/2 nucleus and a static magnetic field in the direction of $\hat{u}=\frac{u}{\left||u|\right|}$, where **u** = (0.137, −0.986, −0.098)*μT*. Hyperfine constants are chosen as *A*_1*x*_ = 0.03, *A*_1*y*_ = −0.64, *A*_1*z*_ = 0.17, *A*_2*x*_ = −0.10, *A*_2*y*_ = 0.0, *A*_2*z*_ = 0.05 all in mT. Fig 5 shows the static magnetic field effect on the singlet quantum yield.

#### Identification of magnetic parameters to minimize quantum yield for a two-proton model

**Fig 6:**
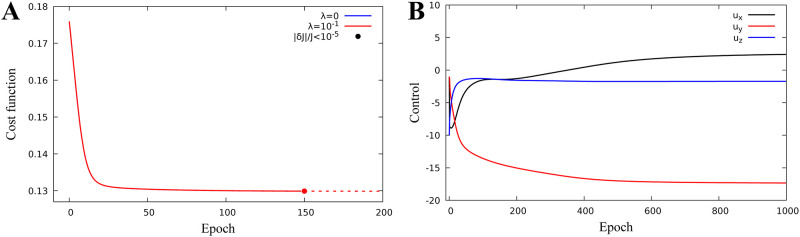
Identification of magnetic parameters for a two-proton model. Iterative method decreases quantum yield to its minimum value J=0.13 represented by the dose-response model. This minimum corresponds to fields (*u*_*x*_, *u*_*y*_, *u*_*z*_) = (2.4, −17.3, −1.7) *μ*T. Negative *u*_*z*_ indicates opposite direction as coordinate frame. Field azimuthal symmetry is lost for this model. **A:** Cost function minimization for different values of the regularization parameter. **B:** Magnetic field parameter evolution.

We consider minimization of the singlet quantum yield for the two-proton problem when *k* = 3, $v=u=\left(u_{x},u_{y},u_{3}\right)\in R^{3}$, i.e. we search for the external magnetic field vector which minimizes the singlet quantum yield. Fig 6A and 6B demonstrate the quantum yield convergence for different regularization parameters and the convergence to the minimizing magnetic field. We observe that regularization is not required for convergence.

#### Identification of hyperfine parameters to minimize quantum yield for a two-proton model

**Fig 7:**
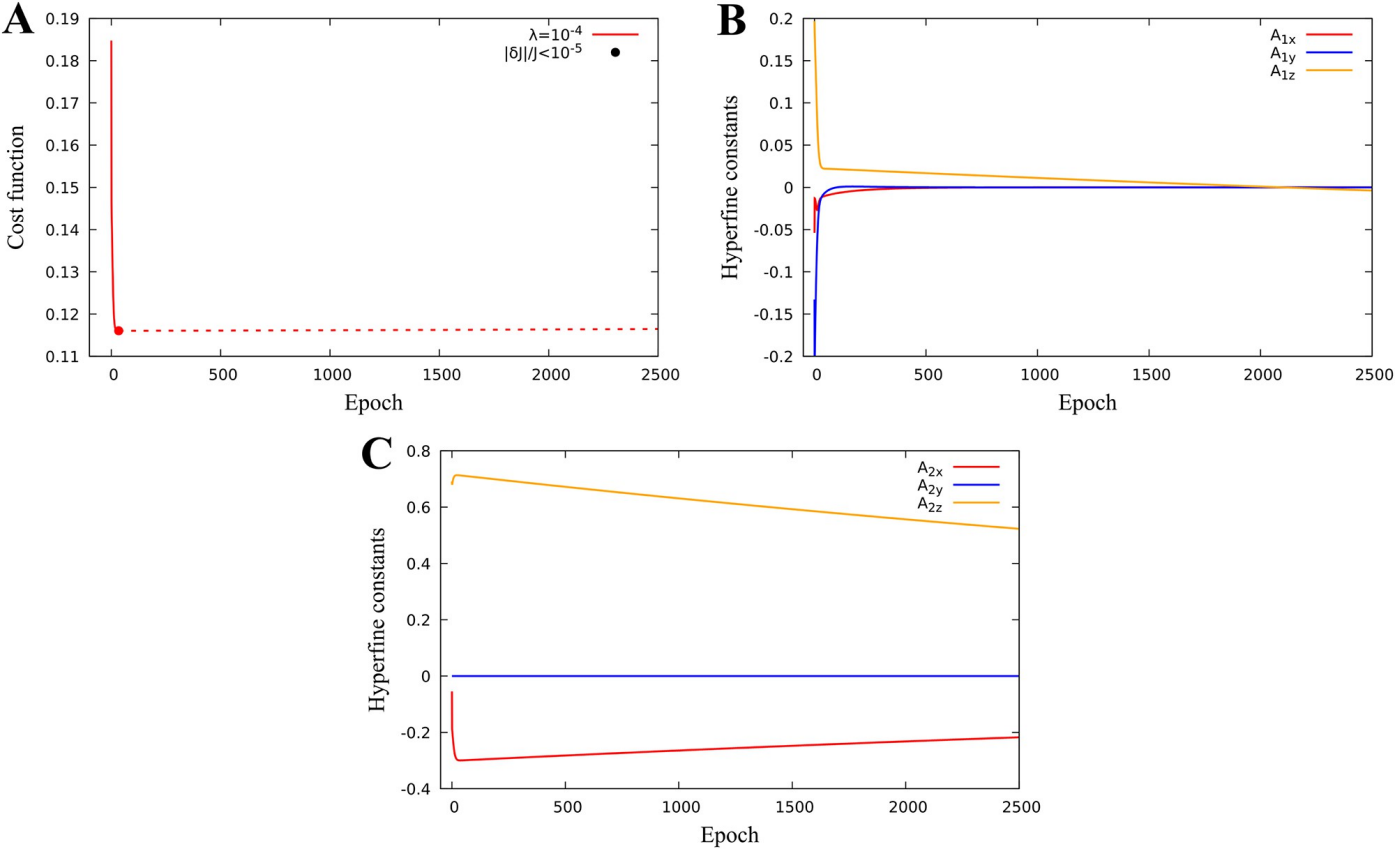
Identification of magnetic parameters for a two-proton model. Iterative method decreases the quantum yield to its minimum value represented by the dose-response model. Optimal values of the hyperfine parameters are *A*_1*x*_ = 0, *A*_1*y*_ = 0, *A*_1*z*_ = −0.004, *A*_2*x*_ = −0.2, *A*_2*y*_ = 0, *A*_2*z*_ = 0.52. **A:** Cost function minimization with regularization. **B:** First proton hyperfine parameter evolution. **C:** Second proton hyperfine parameter evolution.

We consider optimization problem when *k* = 6 and

$$v=\left(A_{1},A_{2}\right)=\left(A_{1x},A_{1y},A_{1z},A_{2x},A_{2y},A_{2z}\right)\in R^{6},$$

i.e. we search for the hyperfine parameters which minimize the singlet quantum yield. Fig 7 shows the minimization with respect to hyperfine parameters with external magnetic field parameter fixed at **u** = (30, 10, 40)*μT*.

#### Identification of combined parameters to minimize quantum yield for a two-proton model

**Fig 8:**
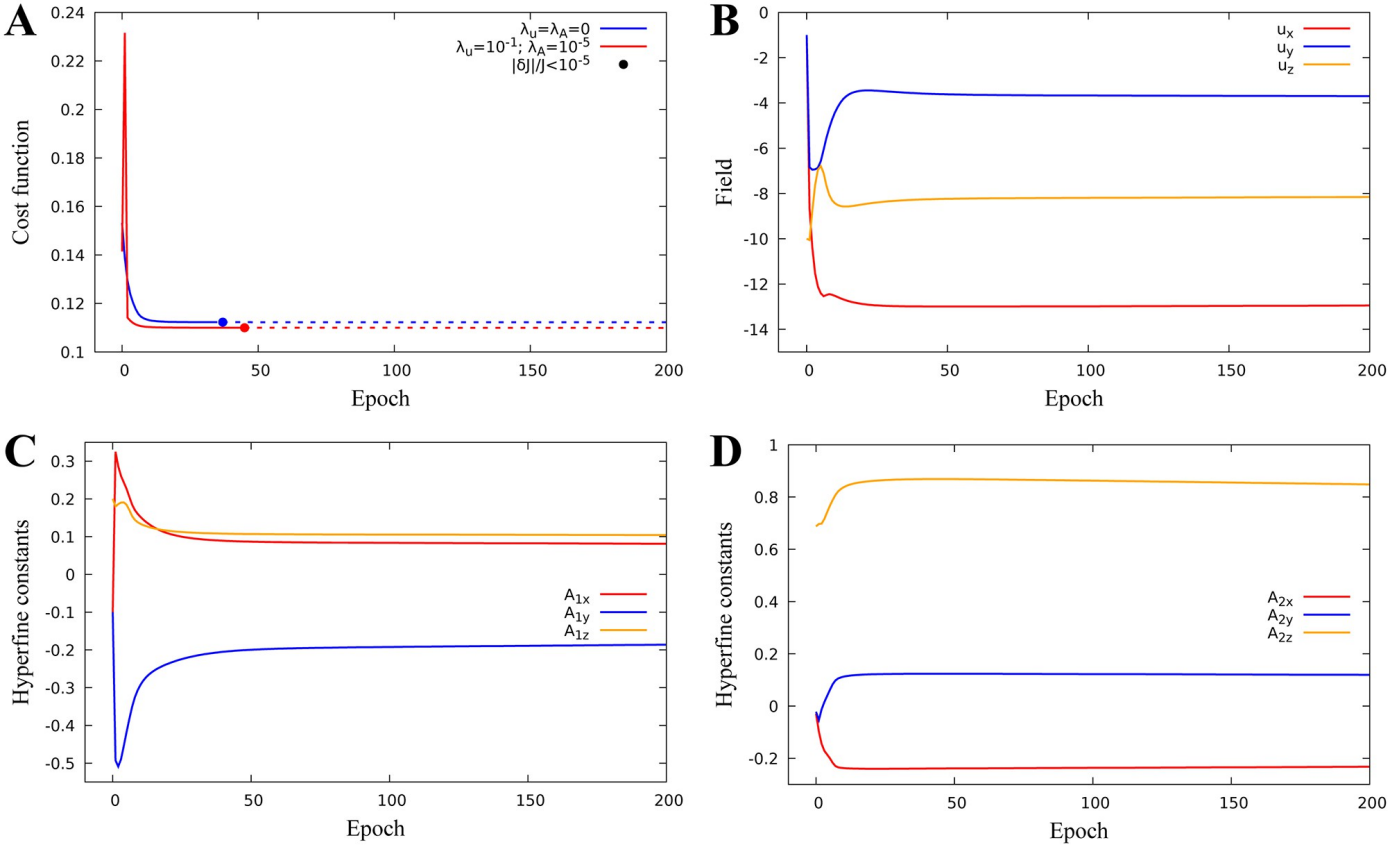
Identification of hyperfine and field parameters for a two-proton model. Quantum yield decreases significantly compared to static response model and reaches the minimum J=0.11 for hyperfine parameters *A*_1*x*_ = 0.081, *A*_1*y*_ = −0.186, *A*_1*z*_ = 0.104, *A*_2*x*_ = −0.232, *A*_2*y*_ = 0.119, *A*_2*z*_ = 0.848 and field (*u*_*x*_, *u*_*y*_, *u*_*z*_) = (−12.9, −3.7, −8.1) *μT*. **A:** Cost function minimization with and without regularization. **B:** Field parameter evolution. **C:** First proton hyperfine parameter evolution.**D:** Second proton hyperfine parameter evolution.

We now consider optimization problem when *k* = 9, $v=\left(u,A_{1},A_{2}\right)\in R^{9}$, i.e. we search for both external magnetic field and hyperfine parameters which minimize the singlet quantum yield. Fig 8A shows the dependence of the minimization of the quantum yield on the regularization parameter. As in the one-proton case, regularization helps faster convergence with respect to function (red graph vs.blue graph in Fig 8A). The algorithm for joint optimization with respect to both magnetic and hyperfine parameters provides a minimizing sequence convergent to a smaller value less than half of the starting quantum yield, which represents an important optimization of the quantum process.

## Discussion and conclusions

In this paper, we consider the problem of identification of the external electromagnetic field and internal hyperfine parameters which optimize the quantum singlet-triplet yield of a simplified radical pair system. We employ the *qlopt* algorithm [13–16] to identify optimal values of 3-dimensional external electromagnetic field vector and 3- or 6-dimensional hyperfine parameter vector which optimize the quantum singlet-triplet yield for the spin dynamics of radical pairs in 8- or 16-dimensional Schrödinger system corresponding to one- and two-proton cases respectively. Numerical results demonstrate that the quantum singlet-triplet yield of the radical pair system can be significantly reduced if optimization is pursued simultaneously for external magnetic field and internal hyperfine parameters. The results may help us understand the structure-function relationship of a complex putative magnetoreceptor to manipulate and enhance quantum coherences at room temperature, and leveraging biofidelic function to inspire novel quantum devices. In particular, the results may provide new routes for weak biomagnetic sensors. The results may represent a crucial step to affirm the direct connection between hyperfine optimization and quantum coherence for more complex radical pair systems.

## Supporting information

S1 Appendix (PDF)
