# Supplementary material for: Optimization of parameters in coherent spin dynamics of radical pairs in quantum biology
Source: PLoS One. 2023 Feb 24;18(2):e0273404. doi: 10.1371/journal.pone.0273404 (PMC9956872; doi:10.1371/journal.pone.0273404)
Supplement: S1 Appendix — (PDF) [file pone.0273404.s001.pdf]

Appendix

Matrix Representation of the Spin Hamiltonian for One-proton Model

$$\mathbf{H}_{hfi} =$$

$$\begin{bmatrix}
\mu_B g \frac{a_z}{4} & 0 & 0 & 0 & 0 & \mu_B g(\frac{a_x}{4} - \frac{a_y}{4}) & 0 & 0 \\
0 & -\mu_B g \frac{a_z}{4} & 0 & 0 & \mu_B g(\frac{a_x}{4} + \frac{a_y}{4}) & 0 & 0 & 0 \\
0 & 0 & \mu_B g \frac{a_z}{4} & 0 & 0 & 0 & 0 & \mu_B g(\frac{a_x}{4} - \frac{a_y}{4}) \\
0 & 0 & 0 & -\mu_B g \frac{a_z}{4} & 0 & 0 & \mu_B g(\frac{a_x}{4} + \frac{a_y}{4}) & 0 \\
0 & \mu_B g(\frac{a_x}{4} + \frac{a_y}{4}) & 0 & 0 & -\mu_B g \frac{a_z}{4} & 0 & 0 & 0 \\
\mu_B g(\frac{a_x}{4} - \frac{a_y}{4}) & 0 & 0 & 0 & 0 & \mu_B g \frac{a_z}{4} & 0 & 0 \\
0 & 0 & 0 & \mu_B g(\frac{a_z}{4} + \frac{a_y}{4}) & 0 & 0 & -\mu_B g \frac{a_z}{4} & 0 \\
0 & 0 & \mu_B g(\frac{a_x}{4} - \frac{a_y}{4}) & 0 & 0 & 0 & 0 & \mu_B g \frac{a_z}{4}
\end{bmatrix}$$

$$\mathbf{H}_z = \begin{bmatrix}
u_z\mu_B g & 0 & (u_x\mu_B g)/2 - (u_y\mu_B gi)/2 & 0 & (u_x\mu_B g)/2 - (u_y\mu_B gi)/2 & 0 & 0 & 0 \\
0 & u_z\mu_B g & 0 & (u_x\mu_B g)/2 - (u_y\mu_B gi)/2 & 0 & (u_x\mu_B g)/2 - (u_y\mu_B gi)/2 & 0 & 0 \\
(u_x\mu_B g)/2 + (u_y\mu_B gi)/2 & 0 & 0 & 0 & 0 & 0 & (u_x\mu_B g)/2 - (u_y\mu_B gi)/2 & 0 \\
0 & (u_x\mu_B g)/2 + (u_y\mu_B gi)/2 & 0 & 0 & 0 & 0 & 0 & (u_x\mu_B g)/2 - (u_y\mu_B gi)/2 \\
(u_x\mu_B g)/2 + (u_y\mu_B gi)/2 & 0 & 0 & 0 & 0 & 0 & (u_x\mu_B g)/2 - (u_y\mu_B gi)/2 & 0 \\
0 & (u_x\mu_B g)/2 + (u_y\mu_B gi)/2 & 0 & 0 & 0 & 0 & 0 & (u_x\mu_B g)/2 - (u_y\mu_B gi)/2 \\
0 & 0 & (u_x\mu_B g)/2 + (u_y\mu_B gi)/2 & 0 & (u_x\mu_B g)/2 + (u_y\mu_B gi)/2 & 0 & -u_z\mu_B g & 0 \\
0 & 0 & 0 & (u_x\mu_B g)/2 + (u_y\mu_B gi)/2 & 0 & (u_x\mu_B g)/2 + (u_y\mu_B gi)/2 & 0 & -u_z\mu_B g
\end{bmatrix}$$

$$\mathbf{K} = \begin{bmatrix} \frac{k_T}{2} & 0 & 0 & 0 & 0 & 0 & 0 & 0 \\ 0 & \frac{k_T}{2} & 0 & 0 & 0 & 0 & 0 & 0 \\ 0 & 0 & \frac{k_T+k_S}{4} & 0 & \frac{k_T-k_S}{4} & 0 & 0 & 0 \\ 0 & 0 & 0 & \frac{k_T+k_S}{4} & 0 & \frac{k_T-k_S}{4} & 0 & 0 \\ 0 & 0 & \frac{k_T-k_S}{4} & 0 & \frac{k_T+k_S}{4} & 0 & 0 & 0 \\ 0 & 0 & 0 & \frac{k_T-k_S}{4} & 0 & \frac{k_T+k_S}{4} & 0 & 0 \\ 0 & 0 & 0 & 0 & 0 & 0 & \frac{k_T}{2} & 0 \\ 0 & 0 & 0 & 0 & 0 & 0 & 0 & \frac{k_T}{2} \end{bmatrix}$$

The representation of the singlet projection operator $\mathbf{P_S}$ in one-proton model (3) is

$$\mathbf{P_S} = \mathbf{D} := \begin{bmatrix} 0 & 0 & 0 & 0 & 0 & 0 & 0 & 0 \\ 0 & 0 & 0 & 0 & 0 & 0 & 0 & 0 \\ 0 & 0 & \frac{1}{2} & 0 & -\frac{1}{2} & 0 & 0 & 0 \\ 0 & 0 & 0 & \frac{1}{2} & 0 & -\frac{1}{2} & 0 & 0 \\ 0 & 0 & -\frac{1}{2} & 0 & \frac{1}{2} & 0 & 0 & 0 \\ 0 & 0 & 0 & -\frac{1}{2} & 0 & \frac{1}{2} & 0 & 0 \\ 0 & 0 & 0 & 0 & 0 & 0 & 0 & 0 \\ 0 & 0 & 0 & 0 & 0 & 0 & 0 & 0 \end{bmatrix} \qquad (37)$$

and

$$\mathbf{P_S} = \mathbf{D} \otimes \mathbf{E}_2,$$

for the two-proton model (7).

Schrödinger System for One-proton Model

For a one-proton model, the Schrödinger system is represented by the following system of 8 ordinary differential differential equations:

$$
\begin{aligned}
\imath\hbar\frac{d\psi_1}{dt} &= \mu_B g\frac{a_z}{4}\psi_1 + \mu_B g(\frac{a_x}{4} - \frac{a_y}{4})\psi_6 + \\
&\quad u_z\mu_B g\psi_1 + \big\{(u_x\mu_B g)/2 - (u_y\mu_B gi)/2\big\}\psi_3 + \big\{(u_x\mu_B g)/2 - (u_y\mu_B gi)/2\big\}\psi_5 - i\frac{k_T}{2}\psi_1 \\
\imath\hbar\frac{d\psi_2}{dt} &= -\mu_B g\frac{a_z}{4}\psi_2 + \mu_B g(\frac{a_x}{4} + \frac{a_y}{4})\psi_5 + \\
&\quad u_z\mu_B g\psi_2 + \big\{(u_x\mu_B g)/2 - (u_y\mu_B gi)/2\big\}\psi_4 + \big\{(u_x\mu_B g)/2 - (u_y\mu_B gi)/2\big\}\psi_6 - i\frac{k_T}{2}\psi_2 \\
\imath\hbar\frac{d\psi_3}{dt} &= \mu_B g\frac{a_z}{4}\psi_3 + \mu_B g(\frac{a_x}{4} - \frac{a_y}{4})\psi_8 + \\
&\big\{(u_x\mu_B g)/2 + (u_y\mu_B gi)/2\big\}\psi_1 + \big\{(u_x\mu_B g)/2 - (u_y\mu_B gi)/2\big\}\psi_7 \\
&-i\frac{k_T + k_S}{4}\psi_3 - i\frac{k_T - k_S}{4}\psi_5 \\
\imath\hbar\frac{d\psi_4}{dt} &= -\mu_B g\frac{a_z}{4}\psi_4 + \mu_B g(\frac{a_x}{4} + \frac{a_y}{4})\psi_7 + \\
&\big\{(u_x\mu_B g)/2 + (u_y\mu_B gi)/2\big\}\psi_2 + \big\{(u_x\mu_B g)/2 - (u_y\mu_B gi)/2\big\}\psi_8 \\
&-i\frac{k_T + k_S}{4}\psi_4 - i\frac{k_T - k_S}{4}\psi_6 \\
\imath\hbar\frac{d\psi_5}{dt} &= \mu_B g(\frac{a_x}{4} + \frac{a_y}{4})\psi_2 - \mu_B g\frac{a_z}{4}\psi_5 + \\
&\big\{(u_x\mu_B g)/2 + (u_y\mu_B gi)/2\big\}\psi_1 + \big\{(u_x\mu_B g)/2 - (u_y\mu_B gi)/2\big\}\psi_7 \\
&-i\frac{k_T - k_S}{4}\psi_3 - i\frac{k_T + k_S}{4}\psi_5 \\
\imath\hbar\frac{d\psi_6}{dt} &= \mu_B g(\frac{a_x}{4} - \frac{a_y}{4})\psi_1 + \mu_B g\frac{a_z}{4}\psi_6 + \\
&\big\{(u_x\mu_B g)/2 + (u_y\mu_B gi)/2\big\}\psi_2 + \big\{(u_x\mu_B g)/2 - (u_y\mu_B gi)/2\big\}\psi_8 \\
&-i\frac{k_T - k_S}{4}\psi_4 - i\frac{k_T + k_S}{4}\psi_6 \\
\imath\hbar\frac{d\psi_7}{dt} &= \mu_B g(\frac{a_z}{4} + \frac{a_y}{4})\psi_4 - \mu_B g\frac{a_z}{4}\psi_7 + \\
&\big\{(u_x\mu_B g)/2 + (u_y\mu_B gi)/2\big\}\psi_3 + \big\{(u_x\mu_B g)/2 + (u_y\mu_B gi)/2\big\}\psi_5 - u_z\mu_B g\psi_7 - i\frac{k_T}{2}\psi_7 \\
\imath\hbar\frac{d\psi_8}{dt} &= \mu_B g(\frac{a_x}{4} - \frac{a_y}{4})\psi_3 + \mu_B g\frac{a_z}{4}\psi_8 + \\
&\big\{(u_x\mu_B g)/2 + (u_y\mu_B gi)/2\big\}\psi_4 + \big\{(u_x\mu_B g)/2 + (u_y\mu_B gi)/2\big\}\psi_6 - u_z\mu_B g\psi_8 - i\frac{k_T}{2}\psi_8.
\end{aligned} \tag{38}
$$
